# A Double-Blind Randomized Trial to Investigate Mechanisms of Antidepressant-Related Dysfunctional Arousal in Depressed or Anxious Youth at Familial Risk for Bipolar Disorder

**DOI:** 10.3390/jpm12061006

**Published:** 2022-06-20

**Authors:** Duncan C. Honeycutt, Melissa P. DelBello, Jeffrey R. Strawn, Laura B. Ramsey, Luis R. Patino, Kyle Hinman, Jeffrey Welge, David J. Miklowitz, Booil Jo, Thomas J. Blom, Kaitlyn M. Bruns, Sarah K. Hamill Skoch, Nicole Starace, Maxwell J. Tallman, Manpreet K. Singh

**Affiliations:** 1 Department of Psychiatry and Behavioral Neuroscience, University of Cincinnati College of Medicine, Cincinnati, OH 45267, USA; honeycdn@mail.uc.edu (D.C.H.); strawnjr@ucmail.uc.edu (J.R.S.); patinolr@ucmail.uc.edu (L.R.P.); welgeja@ucmail.uc.edu (J.W.); blomtj@ucmail.uc.edu (T.J.B.); brunskn@ucmail.uc.edu (K.M.B.); hamillsk@ucmail.uc.edu (S.K.H.S.); tallmamj@ucmail.uc.edu (M.J.T.); 2 Deptartment of Pediatrics Research in Patient Services, Cincinnati Children’s Hospital Medical Center, Pharmacy Research, Cincinnati, OH 45229, USA; laura.ramsey@cchmc.org; 3 Department of Psychiatry and Behavioral Sciences, Stanford Medicine, Stanford, CA 94304, USA; khinman@stanford.edu (K.H.); booil@stanford.edu (B.J.); nstarace@stanford.edu (N.S.); 4 Semel Institute for Neuroscience and Human Behavior, University of California, Los Angeles, CA 90024, USA; dmiklowitz@mednet.ucla.edu

**Keywords:** bipolar risk, hyperarousal, depression, anxiety, adolescent, neuroimaging, escitalopram, pharmacogenetics

## Abstract

Antidepressants are standardly used to treat moderate to severe symptoms of depression and/or anxiety in youth but may also be associated with rare but serious psychiatric adverse events such as irritability, agitation, aggression, or suicidal ideation. Adverse events are especially common in youth with a family history of bipolar disorder (BD) who are at heightened risk for dysfunction in neurobiological systems that regulate emotion and arousal. To further understand this phenomenon, this study will examine (a) baseline risk factors associated with dysfunctional arousal in a sample of youth at high-risk for BD treated with or without an antidepressant, (b) whether antidepressant-related changes in arousal are mediated by changes in prefrontal-limbic circuitry, and (c) whether pharmacogenetic factors influence antidepressant-related changes in arousal. High-risk youth (aged 12–17 years with moderate to severe depressive and/or anxiety symptoms and at least one first-degree relative with bipolar I disorder) will be randomized to receive psychotherapy plus escitalopram or psychotherapy plus placebo. Neuroimaging and behavioral measures of arousal will be collected prior to randomization and at 4 weeks. Samples for pharmacogenetic analysis (serum escitalopram concentration, *CYP2C19* metabolizer phenotype, and *HTR2A* and *SLC6A4* genotypes) will be collected at 8 weeks. Youth will be followed for up to 16 weeks to assess change in arousal measures.

## 1. Introduction

### 1.1. Risk Factors and Putative Mechanisms Underlying Emotional and Physiological Arousal during Antidepressant Treatment

Antidepressants are among the most common medications prescribed to youth in the United States (U.S.) and are used to treat many psychiatric disorders including depression and anxiety disorders [1]. The combined prevalence of depressive and anxiety disorders in U.S. adolescents is approximately 17% [2]. Multiple studies suggest that these medications reduce symptoms [3,4], improve functioning [5], and are generally well tolerated. However, several studies suggest that some youth may experience antidepressant-related psychiatric adverse events [6] such as aggression, psychosis, agitation, suicidal ideation, hypomania, or mania—all behaviors that may be associated with increased emotional arousal [7]. These adverse events are more frequent and potentially more severe in children compared to adults [8,9,10]. We might avoid some of these rare but serious adverse events by identifying youth who are at a greater risk of developing them.

Of particular interest to our study, youth with a family history of BD (i.e., “high-risk” youth) may be predisposed to developing antidepressant-related adverse events. This increased risk may relate to several factors, including increased vulnerability to developing dysfunctional emotional arousal, independent of antidepressant exposure. Indeed, a family history of BD is among the strongest risk factors for developing mood and anxiety disorders in youth [11]. Twin and family studies have provided compelling evidence that having a parent with BD [12,13] is associated with increases in risk for the offspring’s development of disorders of emotional arousal compared with the general population. Moreover, when these offspring develop dysfunctional emotional arousal, their risk of developing BD increases even further [14]. We now also know that multiple genes may moderate the association between having a parent with BD and an offspring’s development of BD [15], and that even healthy youth who have a parent with BD have distinct prefrontal, striatal, and limbic function and connectivity at rest [16], and during emotional [17] and reward processing [18] compared with healthy offspring of depressed and healthy parents. Taken together, these data suggest that neurogenetic vulnerabilities for developing BD precede symptom onset and may progress to manic symptom emergence and the onset of BD.

Antidepressants are commonly used to treat mood and anxiety symptoms [19], which in youth at risk for developing BD may represent prodromal manifestations of BD [8,20]. Therefore, it is difficult to disentangle whether the natural progression of prodromal manifestations and/or antidepressant-related dysfunction leads to the initial onset of manic or hypomanic symptoms. Moreover, whether antidepressants are safe and effective for youth with a family history of BD who present with depressive and anxiety disorders (but with no hypomania or mania symptoms), remains an ongoing clinical dilemma. Further complicating this problem are inconclusive results from intervention studies. Specifically, a few open-label studies in high-risk youth have examined the efficacy of pharmacologic agents including aripiprazole [21], lithium [22], divalproex [23], and quetiapine [24] for a variety of complex mood symptoms, but to date there are no large randomized controlled trials of antidepressants in youth with a family history of BD. Thus, understanding the risk factors for antidepressant-induced adverse events could guide clinicians to either avoid antidepressants or use different monitoring procedures in high-risk youth.

Studies reporting antidepressant-induced adverse events across multiple units of analysis have mechanistically implicated the Arousal construct of the Research Domain Criteria (RDoC) Arousal and Regulatory Systems. RDoC is a research framework for investigating psychiatric disorders that integrates many levels of information (e.g., genomics, neural circuits, behavioral ratings) to explore basic dimensions of functioning that span normal and abnormal human behavior. Arousal facilitates one’s interaction with the environment in a context-specific manner, can be modulated by the physical characteristics and motivational significance of stimuli, and can be regulated by homeostatic drives [25]. In humans and other animals, antidepressants may produce sustained changes in (1) behavioral [6,26] and psychophysiological indexes of arousal including increased emotional, sensory, and motor reactivity [27,28], (2) heart and respiratory rate [29], and (3) structure and function of regions involved in prefrontal-limbic circuits implicated in the generation [30] and regulation of emotional arousal [31]. Indeed, these changes have been observed in individuals with a genetic propensity for rare but serious adverse events from antidepressant exposure (e.g., mania, suicidality) [20]. They also span a range of psychiatric disorders [32], and this cascade of hyperarousal is especially pronounced in youth with a family history of BD [33,34].

With these consideration in mind, our double-blind placebo-controlled study will examine whether specific biomarkers predict the risk of antidepressant-induced hyperarousal and characterize the mechanisms underlying hyperarousal. Several mechanistic hypotheses may explain why some youths are more likely to develop adverse events while others benefit from antidepressants. First, antidepressants may be working to potentiate rather than attenuate a limbic system that may already be primed for reactivity in youth prone to adverse events [35]. Second, youth prone to arousal events may have modified gene expression of monoamine transporters that make it challenging for antidepressants to reach their typical neural targets [36]. Third, in contrast to the expected antidepressant-related decreases in limbic activity, adverse event-related limbic hyperactivity may occur with high antidepressant doses and acute dose escalations [37]. Finally, there are sensitive periods in development (e.g., puberty) when youth may be more vulnerable to antidepressant-related adverse events because of pruning of neural networks [38]. Through a combination of clinical trial design, functional neuroimaging, and pharmacogenetic analysis, we hope to generate data to better inform our understanding of each of these explanations.

### 1.2. Dynamic Functional Networks Subserving Hyperarousal

Neural systems involved in sensory (e.g., amygdala) and emotion regulatory function (e.g., ventral lateral prefrontal cortex, VLPFC) are implicated in emotional arousal. Neural models of adults [39,40] and children [41,42] with symptoms of disrupted emotional arousal across multiple psychiatric syndromes posit broader aberrant interactions between regulatory structures and sensory structures that support the generation and regulation of emotional arousal [43]. These studies suggest that aberrant emotional arousal is associated with dysfunction in a prefrontal-limbic circuit through impaired top-down prefrontal regulation of bottom-up limbic hyperactivity [44,45]. In adults and youth, antidepressants appear to exert their therapeutic effects, in part, by normalizing this aberrant functional activation [46,47,48]. Over time, antidepressants may initially increase [49] and then decrease amygdala activity while increasing prefrontal inhibition of the amygdala. In contrast, one study identified a significant difference in mean activation in the right amygdala among youth with a familial risk for bipolar disorder treated with antidepressants, at-risk youth without antidepressant use, and healthy controls. Specifically, youth at risk for bipolar disorder who were treated with antidepressants and experienced an antidepressant-related adverse reaction had the lowest amygdala activation at baseline [50]. These findings suggest that in youth at risk for bipolar disorder, baseline dysregulation of amygdala activity may be exacerbated by antidepressants and inadequately moderated by prefrontal regulation of amygdala activity, leading to adverse behavioral reactions such as aggression, impulsivity, and irritability [50]. Though replicable findings describe consistent patterns of neural activity and modulation, antidepressants may exert dynamic effects on the brain which require repeated assessment over time.

Other studies have documented antidepressant-related aberrant physiological responses [29,51] and cardiorespiratory decoupling [52] that may be associated with dysfunctional arousal, independent of dosage or symptom severity [53]. Importantly, these patterns of neurobiological dysfunction can persist into adulthood, conferring lifelong vulnerabilities for the development of various psychopathologies [54,55]. Thus, individuals with specific neurobiological characteristics [56] may be at relatively higher risk for antidepressant-related hyperarousal. To date, however, research has been limited to examining risk factors retrospectively, using small samples [57,58], and assessing unitary measures and categorical outcomes. The large, double-blind, placebo-controlled study described herein is designed to address these limitations by using an RDoC organizational framework. 

### 1.3. Escitalopram Pharmacogenetics in High-Risk Youth

Our study will use escitalopram, a selective serotonin reuptake inhibitor (SSRI), which is commonly used to treat depressive and anxiety disorders in children and adolescents [59]. Escitalopram is safe and effective for treating pediatric major depressive and generalized anxiety disorders [60], and is approved by the US Food and Drug Administration (USFDA) for the treatment of major depressive disorder in youth ages 12 and older. Additionally, escitalopram is well-tolerated and cost-effective [61,62]. Given the previously noted risks of developing any psychopathology [54] and/or experiencing a hyperarousal event in response to SSRIs [8,20] among youth at risk for BD, pharmacogenomic assays may provide targeted information about risks for adverse events in these youth (including hyperarousal) during treatment with escitalopram [63]. Modern genetic testing allows for quick genotyping for dozens of genetic polymorphisms which are identified as potential genes of interest in predicting treatment outcomes and rates of adverse events during treatment with escitalopram.

**Figure 1:**
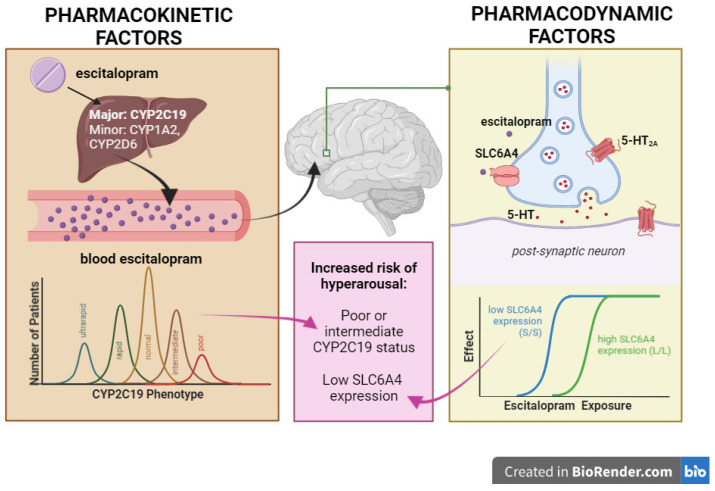
Escitalopram is predominantly metabolized by CYP2C19, and reduced CYP2C19 metabolism has been associated with increased risk of hyperarousal in youth at high risk for bipolar disorder. Youth homozygous for the “short (S)” allele of SLC6A4 exhibit reduced SLC6A4 and greater rates of adverse drug events. Furthermore, 5-HT2A may play a role in escitalopram pharmacodynamics, but its role in the development of hyperarousal or bipolar disorder is less clear. Adapted from Strawn et al. (2021) [80].

In this study, we will obtain a genetic sample from all high-risk youth to identify pharmacokinetic and pharmacodynamic risk alleles. Pharmacokinetic genes of interest include enzymes involved in the metabolism of escitalopram, including members of the cytochrome P450 (CYP) family, such as *CYP2C19*, *CYP2D6*, and *CYP1A2* [33,63,64]. CYP2C19 plays a significant role in the metabolism of escitalopram, and multiple single nucleotide polymorphisms (SNPs) in *CYP2C19* are linked to differences in the rate of escitalopram metabolism [65,66]. Multiple studies suggest that escitalopram dosing regimens should be modified based on *CYP2C19* alleles to achieve comparable therapeutic serum concentrations of escitalopram, as some genotypes confer a greater risk of therapeutic failure or adverse events [64,65,66,67]. Notably, individuals with BD, as compared to those with MDD, have poorer metabolism via CYP2C19, which may predispose them toward antidepressant-induced hyperarousal [68] (see Figure 1). Among adolescents with anxiety disorders treated with escitalopram who experienced hyperarousal, a greater maximum concentration (C_max_) and area under the curve (AUC) of blood serum concentrations of escitalopram was observed compared with adolescents who did not experience hyperarousal symptoms [64].

Other cytochrome P450 genes may also be involved in the metabolism of escitalopram, and some models suggest that pharmacogenomic assays which can integrate the influence of multiple CYP genes may have greater therapeutic utility than a focus on any one CYP gene involved in escitalopram metabolism [69]. For instance, *CYP1A2* polymorphisms contribute to a faster rate of escitalopram metabolism and are linked to higher rates of fatigue, nausea, and vomiting [33], though no escitalopram dosing recommendations are available based on *CYP1A2* alleles. Additionally, smoking induces greater expression of *CYP1A2*, and smoking increases the rate of escitalopram metabolism, a change which correlates with lower serum concentrations of escitalopram [70]. Patient age and weight may also contribute to differences in the rate of escitalopram metabolism, though the clinical significance of these weight- and age-related metabolic differences remains uncertain [71]. Together, these studies suggest that there may be value in examining the combined effects of multiple P450 genes which may contribute to treatment failure or the development of antidepressant-related hyperarousal.

Genetic polymorphisms that influence SSRI pharmacodynamics may also affect treatment outcomes and rates of adverse events. Alleles of interest include polymorphisms in the gene *HTR2A*, which encodes the serotonin receptor 2A subtype (5-HT_2A_), and *SLC6A4*, which encodes the serotonin transporter protein (5-HTT), the primary pharmacologic target of escitalopram. Studies of the influence of *HTR2A* and other serotonin receptor subtypes on adverse reactions in youth have produced mixed results [69,72,73,74,75], and findings related to *SLC6A4* are similarly equivocal [39,63,64,69]. In light of these ambiguities, some researchers have suggested utilizing a combination of risk alleles to produce more consistent pharmacodynamic results [75]. However, a recent study utilizing combinatorial pharmacogenetics in the treatment of depression in adolescents found no advantage in treatment outcomes or rates of adverse events compared to a control group which did not have access to pharmacogenetic testing [76]. These differences in findings may be due to the mix of antidepressants and doses included in the studies, the lack of accounting for specific antidepressant exposure, differences in alleles tested across studies, and other study design considerations including populations studied and the clinical characteristics of participants.

To date, a few pharmacogenetic studies of *HTR2A* or *SLC6A4* have specifically examined the risk of hyperarousal in youth with a familial risk for BD, though their results are inconclusive. An open trial of citalopram in children and adolescents with depressive or anxiety symptoms found that youth who were homozygous for the “short” allele (S/S) of the *SLC6A4* gene exhibited poor antidepressant treatment response for depressive symptoms (but not for anxiety symptoms) compared to youth who were homozygous for the “long” allele (L/L) of *SLC6A4* [77]. A cross-sectional study of youth with at least one parent with BD found antianxiety medication-naïve S/S youth exhibited greater anxiety than L/L youth [78]. Of particular relevance to our study, the S allele of *SLC6A4* increases the risk that high-risk youth will develop arousal symptoms, an effect which was reportedly mediated by greater activity in, and functional connectivity between, the ventrolateral prefrontal cortex (VLPFC) and the right amygdala [79], elegantly intertwining neurogenetic and neurophysiologic systems (see Figure 1). Although more data are needed to support firm conclusions, we hope that our study will advance our understanding of these and other pharmacogenomic interactions and will do so with a larger number of participants in the context of a randomized placebo-controlled trial. Thus, this study will address broader questions about these genes and the underlying mechanisms of antidepressant-related adverse events.

This study will also serve as an initial step to identifying potential biomarkers which might affect antidepressant treatment response and ultimately, might lead to addressing whether very serious antidepressant-related adverse events that result in antidepressant discontinuation or accelerate the development of BD (i.e., via hyperarousal) are preventable in high-risk youth. Our findings will have basic science, translational, and clinical implications. In addition to advancing the neurobiological understanding of arousal and BD within an RDoC framework, focused genetic screening of high-risk youth may lead to scalable and cost-effective innovations in precision medicine.

## 2. Study Protocol

### 2.1. Setting and Study Population

We will recruit 150, 12- to 17-year-old youth with significant depressive or anxiety symptoms, based on responses to Children’s Depression Rating Scale-Revised (CDRS-R) [81] and the Pediatric Anxiety Rating Scale (PARS) [82] clinical interviews and 60, 12- to 17-year-old healthy control youth (ClinicalTrials.gov Identifier: NCT02553161, first posted 17 September 2015). Youth with significant depressive or anxiety symptoms will be included in our study, and psychotherapy and the study medication (escitalopram) will be provided to study participants to treat their depressive and/or anxiety disorders. Although participants’ DSM-5 diagnoses will be assessed using KSADS, we will not require a specific diagnosis for inclusion and will only exclude participants with the following diagnoses: bipolar I or II disorder, autism spectrum disorders, obsessive compulsive disorder, post-traumatic stress disorder, Tourette’s disorder, or any psychotic disorder including schizophrenia. Our inclusion and exclusion criteria are summarized in Table 1. Healthy controls will receive behavioral, neural, and physiological assessments at baseline only. First-degree relatives will be assessed using the Structured Clinical Interview for Diagnostic and Statistical Manual (SCID) [83] and the Family History-Research Diagnostic Criteria (FH-RDC). We will also compare our findings to those of a recently published double-blind, placebo-controlled trial using the same neuroimaging task and the same medication, but in youth *without* a family history of BD [84].

### 2.2. Randomization

High-risk youth will be randomized to receive double-blinded treatment with psychotherapy plus escitalopram (MED) vs. psychotherapy plus placebo (No MED) in a 2:1 ratio using block randomization with a block size of six. Randomization will be stratified within sites with respect to diagnoses (anxiety only, depression only, or anxiety and depressive symptoms), family history load (i.e., ≥3 first- or second- degree relatives with bipolar disorder vs. 1–2 first- or second-degree relatives with bipolar disorder), and the presence (vs. absence) of concomitant stimulant or alpha-2 agonist treatment. 

We selected psychotherapy (No MED) as the most ethically reasonable comparator for youth presenting with clinically significant symptoms of depression or anxiety. All participants (No MED and MED) will be assigned a study-trained therapist who will provide hour-long, up to weekly, individual cognitive-behavioral therapy or family-focused therapy (FFT) for high-risk youth [87,88]. FFT is a semistructured treatment that is protocol-driven and incorporates psychoeducation about mood and stress vulnerability models for depression/anxiety, structured communication training among family members, and problem-solving skills. Sessions will be weekly for the first 4 weeks, then participants will be given the option to continue meeting weekly or once every two weeks for an additional 12 weeks.

### 2.3. Medication Dosing and Accountability

High-risk youth in the MED condition will be treated with escitalopram and monitored by a child and adolescent psychiatrist who is blinded to treatment assignment. Each high-risk participant will be assessed weekly for the first 4 weeks, then once every two weeks until 16 weeks. Escitalopram will be initiated at 5 mg daily for 1 week, then titrated to 10 mg daily for 1 week, then titrated to a target dose of 20 mg daily (maximum dose 30 mg/day). Titration will proceed no faster than 5 mg/week. Clinicians will carefully monitor participants for treatment response based on symptom severity rating scales of anxiety (PARS), depression (CDRS-R), (hypo)mania (Young Mania Rating Scale) [89], and the emergence of dysfunctional emotional arousal using the clinician-version of the Pediatric Adverse Events Rating Scale (PAERS) [90] and the parent-rated Treatment-Emergent Activation and Suicidality Assessment Profile (TEASAP) [91]. Escitalopram (or placebo) will be discontinued if there is a 2-step worsening on the Clinical Global Impression-Severity of Activation (CGI-SA) scale [91] compared with baseline and a Children’s Global Assessment Scale (CGAS) [92] score of <55.

Adherence, based on pill count, will be obtained at each visit. First, participants will receive weekly clinical follow-ups during dose titration, then clinical follow-ups every two weeks until week 16 to closely monitor for treatment adherence. Blood samples for analysis of serum escitalopram and desmethylescitalopram concentrations will be drawn at 8 weeks or at early termination if sooner than week 8. We aim to recruit medication-naïve youth but recognize that some high-risk youth who are seeking treatment for significant symptoms may be exposed to prior psychotropic medications. We will consider the role of past medications in our analysis of primary outcomes, and participants will be allowed to continue stable doses of concomitant stimulants and alpha-2 agonists. 

### 2.4. Primary Clinical Outcome and Neural and Pharmacogenetic Mediators of Outcome

Our primary clinical arousal outcome will be the highest score on the arousal-based items of the clinician PAERS. The PAERS will be administered to all youth at baseline, and to high-risk youth weekly for the next 4 weeks then once every two weeks until week 16. We will explore subgroups of arousal due to worsening symptom severities of mania, anxiety, depression, psychosis, suicidality, and anxiety using clinician-, self-, and parent-reported (e.g., TEASAP [91]) measures of emotional reactivity and lability, and reaction times during the Continuous Performance Task with Emotional and Neutral Distracters (CPT-END) as secondary predictors of dysfunctional emotional arousal [93]. Heart rate, respiratory rate, and galvanic skin response will be collected while the participants undergo an MR scan during rest and during presentation of emotionally arousing stimuli as ancillary objective measurements of physiological arousal to support our neural predictions. Based on prior studies [29,51], we hypothesize that high-risk youth who develop antidepressant-related hyperarousal will exhibit, at baseline, greater physiological responses to emotionally arousing stimuli compared to high-risk youth who do not develop antidepressant-related hyperarousal. We will compare arousal in the MED and No MED conditions at its highest point between 4 and 16 weeks, which we believe will result in the most meaningful comparison across groups that also accounts for the possibility that some youth may improve with intervention.

#### 2.4.1. Neural Mediators

Our main neural outcomes are defined by prior studies implicating amygdala and VLPFC activation. We will evaluate activation in these regions during the emotional-neutral contrast of the Continuous Performance Task with Emotional and Neutral Distracters (CPT-END) task, a task of sustained attention with emotional and neutral pictures which we have used in our prior work examining prefrontal-limbic connectivity [94,95]. To assess the complex prefrontal-limbic circuit mechanisms that subserve antidepressant-related arousal both intrinsically at rest and during emotional arousal, youth will complete a resting state scan followed by the CPT-END in the MR scanner. Connectivity during the CPT-END and at rest will also be assessed. MRI data will be acquired at baseline for high-risk youth and controls, and at 4 weeks in high-risk youth using a 3.0 Tesla GE whole-body MRI scanner located at Cincinnati Children’s Hospital Medical Center Imaging Research Center and Stanford’s Richard M. Lucas Center for Imaging. An additional MRI scan will be acquired in any high-risk youth who develops antidepressant-related dysfunctional emotional arousal prior to 4 weeks, or up to one additional scan after 4 weeks if a hyperarousal event occurs between 4–16 weeks.

**Figure 2:**
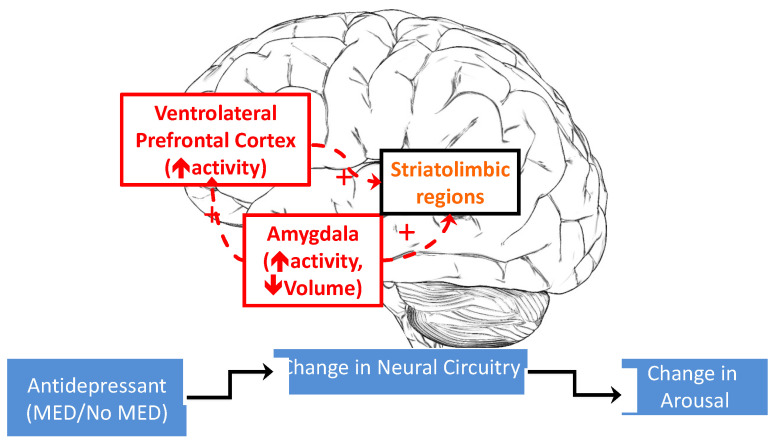
Conceptual framework for the formal evaluation of neural mechanisms of change as mediating medication effect on the development of hyperarousal. Legend: ↑ = increase; ↓ = decrease; + = hyperconnectivity.

Participants will complete two fMRI runs of the emotionally arousing CPT-END task, in which whole-brain images (volumes) will be acquired every 3 s using a T2*-weighted gradient-echo echo-planar imaging pulse sequence. We will explore whether high-risk youth who do not develop antidepressant-related arousal benefit from antidepressant treatment, which has been documented to reduce amygdalar activation [96]. For those high-risk youth who are not exposed to escitalopram, we predict that there will be effects on limbic activation and connectivity due to a combination of psychotherapeutic effects [97], trait vulnerability to prefrontal-limbic dysfunction [35], and a natural propensity toward symptom progression [98]. We hypothesize that early change (baseline to 4 weeks) in the level of amygdala hyperactivity will predict the level of subsequent arousal (highest after the 4-week scan) and that the correlation between changes in neural circuitry and the level of subsequent arousal is stronger in the MED condition compared to the No MED condition (Figure 2).

#### 2.4.2. Pharmacogenetic Mediators

Our main pharmacogenetic-related mediators are *CYP2C19* metabolizer phenotype and serum escitalopram level. At 8 weeks or discontinuation of study participation (if earlier), we will obtain a sample for pharmacogenetic testing with the GeneSight Psychotropic assay. *CYP2C19* metabolizer phenotypes will be determined using Clinical Pharmacogenetics Implementation Consortium (CPIC) guidelines [99] and classified as normal (NM), rapid (RM), ultrarapid (UM), intermediate (IM), or poor (PM) metabolizer phenotypes. We will also perform a blood draw at 8 weeks or at early termination to determine serum escitalopram levels via mass spectrometry. Serum escitalopram concentration, *CYP2C19* metabolizer phenotypes, and medication adherence data will be integrated and modelled using MWPharm++ to determine pharmacokinetic parameters such as the maximum serum concentration (C_max_), the 24 h area under the curve (AUC_24_), the elimination half-life (t_1/2_), and clearance (CL) of escitalopram. Genetic testing reports will identify alleles in other genes as well (e.g., *CYP2D6*, *CYP2C9*, *CYP1A2*, *SLC6A4*, and *HTR2A*) and we will explore associations between these alleles and outcomes such as treatment efficacy and rates of adverse events. Based on similar studies of escitalopram in youth [64,65], we predict that high-risk youth with reduced *CYP2C19* metabolizer phenotypes will have higher C_max_ and/or AUC_24_ and higher rates of arousal symptoms than high-risk youth with normal or elevated *CYP2C19* metabolizer phenotypes.

### 2.5. Hypotheses and Analytic Plan

Our core analytic strategy is mixed-effects modeling [100] utilizing baseline and highest levels of arousal assessed between 4–16 weeks as our primary outcome. In the mixed effects analysis, change in arousal (baseline to post, where post is defined as the highest level of arousal recorded between 4 and 16 weeks) will be the outcome predicted by treatment status (MED vs. No MED); escitalopram levels and neural changes will be the primary mediators. This method was chosen for improved precision (power) and better handling of missing data compared to approaches that use single change scores or analysis of covariance. We will use the MacArthur framework [101,102], which will be embedded in our mixed-effects modeling, to investigate treatment effect mediators, moderators, and nonspecific predictors. We have chosen this framework as the preferred approach to define and test mediation because this framework allows for stronger inferences regarding temporal relations, and the research question posed has significant implications for clinical decision-making [102].

Power estimation for mediation hypotheses was conducted using Monte Carlo simulations based on a single longitudinal mixed-effects model. Previous studies showed effect sizes ranging from d = 0.6 to 1.8 for the effect of antidepressant medication on neural outcomes [20,49]. We assumed a conservative effect size of d = 0.5 given that we will measure changes in neural outcomes early on (4 weeks). We allowed a moderate difference across the two sites (d = 0.6 vs. d = 0.4, on average d = 0.5). Under this scenario, with the initial N = 150 (Stanford:50 MED, 25 No MED; UC:50 MED, 25 No MED), the estimated power to detect the treatment effect (MED vs. No MED) on change in amygdala hyperactivity is 0.82 (two-tailed, alpha = 0.05).

### 2.6. Limitations

The proposed study will gather data on high-risk youth only during a 16-week period, so this study cannot determine whether there are long-term risks or delayed effects of antidepressant exposure in high-risk youth far beyond the duration of the study. We expect that most children who enroll in our study may still live with their first-degree relatives with BD, so our study cannot easily differentiate between the mental health risks posed by heritable genetic factors as opposed to the stress of living with a family member (especially a parent) with a serious psychiatric disorder. Treatment adherence will be well-tracked, but achieving reliable medication adherence in adolescents remains challenging. Even though minor lapses in medication adherence may have relatively little effect on clinical or neuroimaging outcomes, missed or extra doses may introduce significant noise into pharmacokinetic data. For example, serum escitalopram levels can vary greatly when doses are missed in the week before blood samples are drawn. Furthermore, youth who experience hyperarousal symptoms may be more distressed and consequently less likely to participate in some inherently stressful aspects of the study, such as entering the MR scanner or providing a cheek swab or blood sample for pharmacogenetic analysis. Finally, we expect that most youth enrolled in this study will be medication-naïve, but some study participants may be allowed to take concomitant psychostimulants or alpha-2 agonists before and during the study, and this may also confound results.

## 3. Impact of the Study

This study will be highly innovative and may have important implications for developing integrative neurobehavioral models of dysfunctional emotional arousal and advancing our understanding of the neuropathophysiology of bipolar disorder. It also has the potential to advance our understanding of the role of antidepressants in dysfunctional emotional arousal by identifying clinical, neural, and pharmacogenetic risk factors, which may ultimately improve treatments for emotional dysregulation in youth. This study will be the first rigorously designed prospective study to examine data from clinical, neuroimaging, physiologic, and pharmacogenetic sources to understand antidepressant-related dysfunctional emotional arousal in youth. Assessments proposed have not been previously applied to examine the mechanisms underlying antidepressant-induced dysfunction in emotional arousal, a clinically significant problem for which there is very limited evidence. Most studies to date have examined antidepressant-related adverse events retrospectively, observationally, and categorically (presence/absence of an adverse event) in youth treated for single and heterogeneous disorders [6]. Here, we aim to elucidate factors involved in antidepressant-related adverse events in youth with a focus on identifying levels of dysfunction associated with these events in specific neurophysiologic constructs that cut across traditional diagnostic categories. Such an innovative approach is consistent with the RDoC framework which aims to capture variations in different units of analysis and in samples with inherent dimensionality in clinical presentation with a range of symptoms. Finally, this study has the potential to advance not only the neurobiological understanding of bipolar disorder, but also the neurophysiologic and pharmacologic mechanisms of arousal more generally. We hope our results lead to actionable improvements in the care of this very vulnerable population and ultimately, will allow for the prevention of bipolar disorder in at-risk youth.

## 4. Ethics Statement

The study will be conducted according to the guidelines of the Declaration of Helsinki and is approved by the Institutional Review Boards of the University of Cincinnati and Stanford University. Additionally, an NIH-commissioned data safety monitoring board will oversee the conduct of the study. ClinicalTrials.gov Identifier: NCT04623099.

## Figures and Tables

**Table 1:** Inclusion and exclusion criteria for study participants.

Inclusion Criteria: High-Risk Youth	Inclusion Criteria: Healthy Controls
1. Aged from 12 years, 0 months to 17 years, 11 months	1. Aged from 12 years, 0 months to 17 years, 11 months
2. At least one parent, step-parent, or guardian with whom the subject lives is willing to participate in research sessions	2. At least one parent, step-parent, or guardian with whom the subject lives is willing to participate in research sessions
3. The child and relative(s) are able and willing to give written informed assent/consent to participate, respectively	3. The child and relative(s) are able and willing to give written informed assent/consent to participate, respectively
4. At least one first-degree relative with bipolar I disorder as assessed by the Structured Clinical Interview for DSM (SCID) [83], the Kiddie Schedule for Affective Disorders and Schizophrenia (KSADS-PL) [85], and the FH-RDC [86]	4. No personal psychopathology (except specific phobias)
5. Evidence of current, significant depressive or anxiety symptoms as determined by a current Childhood Depression Rating Scale-Revised (CDRS-R) [81] score > 35 and/or a current Pediatric Anxiety Rating Scale (PARS) score > 15 [82].	5. No family history (first- or second-degree) of any mood or psychotic disorders
**Exclusion Criteria: All Participants**	
1. Any history of syndromal bipolar I or II disorder (i.e., history of mania, mixed episode, or major depression with hypomania)	
2. A history of previous antidepressant exposure	
3. A DSM-5 diagnosis of autism spectrum disorders, obsessive compulsive disorder, post-traumatic stress disorder, Tourette’s disorder, or any psychotic disorder including schizophrenia	
4. Evidence of intellectual disability (IQ < 70) as determined by the Weschler Abbreviated Scale of Intelligence (WASI; Psychological Corporation, 1999)	
5. Comorbid neurologic diseases such as seizure disorder	
6. Drug or alcohol abuse or dependence disorders in the 4 months prior to study recruitment, although a lifetime history of substance or alcohol disorders could be present if the child has been abstinent for at least 6 months	
7. Evidence of an unstable medical or psychiatric disorder that required immediate hospitalization or other emergency medical treatment	
8. A positive pregnancy test	
9. Any contraindication for MRI, including metal in the body related to an injury or surgery (e.g., surgical clips, metal fragments in the eyes), piercings that could not be removed, braces, or permanent retainers	

## Data Availability

Not applicable.
